# Assessment of the effect of pre-conception care on preventing mother-to-child transmission of HIV in Nyeri County

**DOI:** 10.11604/pamj.2024.47.144.37196

**Published:** 2024-03-27

**Authors:** Lulu Nazi Ndapatani, Job Mapesa, Emily Muchina

**Affiliations:** 1 Kenya Methodist University, 45240 - 00100, Nairobi, Kenya

**Keywords:** Mother-to-child transmission, pre-conception care, human immunodeficiency virus, Nyeri County

## Abstract

**Introduction:**

the provision of essential preconception care services for HIV-positive pregnant women is crucial to prevent HIV transmission to infants. This includes pregnancy intention screening services, adequate viral load monitoring and suppression before conception, and necessary nutritional support. In Nyeri County, the prevalence of Mother-to-Child Transmission (MTCT) of HIV is 5.3%, which is higher than the global threshold of 5%. This study aims to evaluate the impact of pre-conception care services in preventing HIV transmission to infants in Nyeri County. The study objectives are to assess the utilization of pre-conception care services among HIV-positive women, specifically focusing on pregnancy intention screening, viral load monitoring and suppression, and access to nutritional assessment services before pregnancy. Additionally, the study aims to investigate the relationship between the provision of pre-conception care services and infant HIV outcomes.

**Methods:**

this cross-sectional retrospective descriptive study employed stratified sampling to select eight level 4 and level 5 hospitals in Nyeri County. The target population consisted of HIV-infected women seeking postnatal care in these facilities, with a sample size of 252 women who had HIV-exposed infants under two years old and were receiving post-natal care at the respective hospitals. Sociodemographic characteristics, including age, marital status, and education level, were collected. Data analysis involved both descriptive and inferential statistics.

**Results:**

our findings revealed that only 34.2% of HIV-positive women seeking postnatal care had received information or services related to pregnancy intention screening, a crucial aspect of pre-conception care. Almost half (46.4%) of the women who participated in the study had undergone viral load measurements before pregnancy, which is another critical component of preconception care. Additionally, 85.6% of these women had received nutritional services during pregnancy from their healthcare providers. Interestingly, all women who received any pre-conception care services reported that their infants were alive and tested HIV-negative.

**Conclusion:**

preconception care is crucial in preventing mother-to-child transmission of HIV. Efforts should be made to ensure that all HIV-infected women planning to conceive have access to preconception care services.

## Introduction

Pre-conception care refers to a set of healthcare interventions provided to women and couples before they conceive. The goal is to enhance their overall health, reduce behaviors, and address individual and environmental factors that could negatively impact maternal and child health outcomes. Prevention of mother-to-child transmission (PMTCT) involves offering a range of services to women of reproductive age who are either at risk of acquiring HIV or already living with HIV. The aim is to maintain their health and prevent their infants from becoming infected with HIV. Mother-to-child transmission (MTCT) of the Human Immunodeficiency Virus (HIV) is a major global concern, and efforts are being made to eradicate this transmission by implementing community-sensitive strategies [1]. Sub-Saharan Africa, which is home to an estimated 19.6 million people living with HIV, of whom 56% are women, bears the burden of over 50% of the world's HIV cases [2]. Integrated HIV services emphasize the significance of preconception care to control unplanned pregnancies and enhance health outcomes. Initiating Antiretroviral Therapy (ART) before pregnancy offers the lowest MTCT risk, with the duration of ART correlating directly with MTCT risk reduction [3]. The pivotal factor in infant survival is viral suppression, a more attainable goal for women who commence ART before conception [3]. Kenya grapples with an 8.3% mother-to-child transmission rate, and evidence suggests that implementing preconception care could potentially reduce HIV transmission from mother to child to the World Health Organization's recommended global threshold of 5% [4]. Nyeri County presents a distinctive context, with women exhibiting a higher HIV prevalence rate (5.3%) than men (1.8%), underscoring the heightened risk of women of reproductive age contracting HIV [5].

Given the desire of these women to build families in the future and the elevated MTCT rate (5.3%), preconception care emerges as a critical service [5]. Local HIV programs in the county advocate adhering to Kenya's HIV guidelines, which include integrating preconception care services into routine HIV service delivery and ensuring the health and viral suppression of HIV-positive women before conception. In Nyeri County, non-governmental organizations have invested approximately 1.65 million US dollars in biomedical interventions, yet gaps in treatment coverage persist, with PMTCT services reaching only around 84% coverage, and the HIV testing services provided are considered suboptimal [6]. In 2018, the number of PMTCT projects implemented in biomedical programs was estimated at four, a slightly lower figure than in other counties [6]. There is a glaring lack of data regarding the effectiveness of programs like preconception care services within HIV service delivery and their outcomes in Nyeri County. Research has unequivocally shown that offering preconception care services to HIV-positive women of reproductive age significantly reduces the risk of MTCT [7]. However, there is a gap in knowledge and information regarding the status and effectiveness of these services, posing a significant challenge to maternal and child health outcomes in Nyeri County. The study aims to assess the effectiveness of pre-conception care services in reducing mother-to-child transmission (MTCT) of HIV in Nyeri County. The objectives include evaluating the prevalence of planned pregnancies and awareness of prenatal care services among HIV-positive women, examining the utilization of preconception care services (including antiretroviral therapy initiation before pregnancy) to mitigate MTCT risk, investigating the association between ART initiation before conception and viral suppression as a crucial factor in infant survival, and analyzing the distinctive context of Nyeri County with a higher HIV prevalence among women and its implications for preconception care needs.

## Methods

**Study design:** this was a cross-sectional retrospective descriptive study; it has previously been used effectively to research the effectiveness of PMTCT in HIV programs in rural western Kenya [8]. This design was considered suitable for our study.

**Setting:** the study was conducted in Nyeri County, located approximately 153 kilometers north of Nairobi. Participants in this study were women receiving post-natal PMTCT services in Nyeri County who had tested positive for HIV.

**Participants:** we targeted public hospitals at levels 4 and 5 in 5 Nyeri Sub-Counties, where most women seek PMTCT care. Inclusion criteria specified that participants must be HIV-positive women with HIV-exposed infants under the age of 2 years, receiving post-natal care in the HIV clinic within the health facility, and who had provided written consent to participate. Stratified sampling was employed, with the strata consisting of HIV-infected post-natal clients attending clinic visits in the respective hospitals in Nyeri. In 2018, there were 435 women in Nyeri County receiving post-natal PMTCT services [9].

**Variables:** the study focused on several variables, including the provision of pre-conception care services (such as pregnancy intention screening, viral load testing, and nutritional services), as well as infant HIV outcomes.

**Data sources:** primary data sources included responses obtained from a structured questionnaire administered to HIV-infected women with HIV-exposed infants under 2 years old who were receiving post-natal care in health facilities. Primary data encompassed information on pregnancy intention screening services, nutritional services, and infant HIV testing. Secondary data comprised viral load measurements and anthropometric data.

**Bias:** to minimize design and selection bias, a well-planned research approach was implemented. To counteract order effects bias, the survey questions were randomized, and a pilot test was conducted to assess the effectiveness of the survey instrument.

**Sample size:** given a population size of 435 and a population proportion of 0.5, with an expected error of 4%, the sample size was determined using Cochran's standard formula, typically employed when the population is finite. The sample size for this study consisted of 252 respondents.

**Quantitative variables:** all variables under investigation in this study were quantitative, including pregnancy intention screening, viral load measurements, nutritional services, and infant HIV testing.

**Statistical methods:** the coded questionnaire was transferred to SPSS version 26. Descriptive statistics were performed to illustrate the basic characteristics of the study population. Frequencies were calculated for categorical variables and chi-square values were determined for demographic, clinical, and viral loads. During the analysis in SPSS, missing data were excluded case-wise to enhance the quality and reliability of the analysis.

**Ethics:** ethical approval was obtained from the Kenya Methodist University Institutional Scientific Ethics Review Committee (KeMU/SERC/PHT/56/2019), and the study also received approval from the Kenya National Commission for Science, Technology, and Innovation (NACOSTI) permit No. 995531. Additionally, Nyeri County granted research authorization (REF: CGN/Health/HRM/5/Vol.II).

## Results

**Participants:** for this study, a response rate of 77% was attained from a sample size of 252 HIV-positive pregnant women who were receiving preconception care services in medical institutions in Nyeri County at the time of the sampling (Table 1).

**Table 1:** proportionate sample size from each hospital in Nyeri County

Hospital name	Percentage of HIV-infected postnatal care (PNC) clients receiving care	Sampled number of HIV-infected PNC clients receiving care
Nyeri County referral hospital	25.5	64
Karatina sub-county hospital	27.4	69
Mukurweini hospital	15.4	39
Othaya hospital	13.3	34
Naromoru hospital	10.1	25
Mt Kenya hospital	8.3	21
**Total**	100	252

**Descriptive data:** in all, 91.8% (n=197) of respondents fell within the 18-40 age bracket, which is representative of the wider demographic groupings seen in Kenya and throughout Africa. Most respondents were between the ages of 18 and 40, highlighting the significance of this study as it directly affects women who can have children. The respondents had at least an elementary level of education. Just 14.9% (n=29) of all participants, however, had completed tertiary education while 33.9% (n=66) were either single or divorced/separated, and 66.1% (n=129) were married (Table 2).

**Table 2:** demographic, clinical, and viral load characteristics of participants

Characteristic	n	Percent	χ (P-value)
**Age categories of respondents**			
18-30 years	77	39.5	7.092 (0.131)
31-40 years	102	52.3	
41-50 years	16	8.2	
**Total**	195	100	
**Level of education**			
Primary	79	40.5	7.290 (0.121)
Secondary	87	44.6	
Tertiary	29	14.9	
**Total**	195	100	
**Marital status**			
Single	51	26.2	
Married	129	66.1	
Separated/divorced	15	7.7	
**Total**	195	100	
**Did you plan for the pregnancy?**			
Yes	65	33.7	4.422 (0.011)
No	128	66.3	
**Total**	193	100	
**Did you receive any information from a healthcare professional?**			
Yes	65	34.2	
No	125	65.8	
**Total**	190	100	
**Did you monitor your viral load before pregnancy?**			
Yes	65	34.2	
No	125	65.8	
**Total**	190	100	

**Demographic, clinical, and viral load characteristics of participants:** according to (Table 2), only 33.7% of respondents had planned for their pregnancy, showing that they had made a deliberate decision to conceive, while 66.3% had not. In addition, just 34.2% of respondents said their healthcare providers informed them about prenatal care, as opposed to 65.8% of respondents who said they did not receive any prenatal care information from their healthcare providers. A cross-tabulation analysis of educational level and planned pregnancy found a Chi-square value of 4.422 and a p-value of 0.011, which is less than the standard alpha value of 0.05. The result is statistically significant, demonstrating that the respondent's educational level and the likelihood of planned conception are linked signifying that the respondent's educational level has an impact on whether they intend to become pregnant or not. In summary, pregnancy intention screening for the respondents was low.

About 65.8% of respondents did not assess their viral load before conception, compared to 34.2 percent of respondents who did (Table 2). Ninety percent (n=81) of the research participants whose viral loads were assessed had zero or undetectable viral levels. 8.6%, had viral loads of 1000 or more. In addition, 71.3 (n=139) percent of the respondents were taking ARVs before becoming pregnant, compared to 28.7 (n=56) percent who were not. The 28.5 percent who are not taking ARVs run a higher risk of passing the virus on to their children. 21.6 (n=30) percent of individuals on ARVs before pregnancy had been on them for less than a year, 41.7 (n=58) percent had been on them for 2 to 4 years, and 36.7 (n=51) percent had been on them for more than 5 years. The longer HIV-infected women who wish to become pregnant take their ARVs, the greater their odds of decreasing HIV MTCT.

**Nutritional services, health status, and counseling among participants:** in Nyeri County, 85.6 (n=167) percent of respondents received nutritional services from their healthcare providers, while 14.4 (n=28) percent did not. The weights, heights, and BMIs of those who received nutritional services were taken to determine how healthy they were. 76.1 (n=127) percent of the respondents were normal while 22.8 (n=38) percent were overweight (Table 3). Only 38.9 (n=76) percent of those who received nutritional assistance received nutrition counseling and advice, while 61.1 (n=119) percent did not receive any counseling or advice. In addition, only 9.8 percent of respondents who had received nutritional services received nutritional supplements such as folic acid, which is necessary for pregnant moms, while 90.2 (n=176) percent did not. 85.6 (n=166) percent of the respondents received nutritional services from their healthcare providers in Nyeri County.

**Table 3:** nutritional services offered

Received nutritional services	n	Percentage
Yes	167	85.6
No	28	14.4
**Total**	195	100
**For the respondents that answered, yes, to having received nutritional services (weights, BMIs, and Heights were used to determine health status)**		
Nutrition status		
Under-weight	2	1.1
Normal	127	76.1
Over-weight	38	22.8
**Total**	167	100
**Received any nutritional counseling services in preparation for conception**		
Yes	76	38.9
No	119	61.1
Total	195	100
**Received any nutritional supplements before conception**		
Yes	19	9.8
No	176	90.2
**Total**	195	100

**Infant survival, ARV prophylaxis, and HIV testing outcomes among respondents:** according to (Table 4) percent indicated that the participant's children had taken ARV prophylaxis at birth. Furthermore, 55.4 (n=108) percent of respondents said their children are still on ARV prophylaxis, while 43.6 (n=86) percent said their newborns had stopped ARV prophylaxis. On the HIV status of the infants at the time of the study, 96.9% (n=188) of the respondents said their infants had been tested for HIV, and only 3.2 (n=6) percent said they had not. 100 (n=190) percent of the infants who had been tested were HIV-negative.

**Table 4:** infant HIV outcomes

Is Infant alive	n	Percent
Yes	194	100
No	-	-
**Total**	194	100
**Did your child receive ARV prophylaxis after delivery?**		
Yes	193	99.5
No	1	0.5
**Total**	194	100
**Is your child still taking ARV prophylaxis?**		
Yes	108	55.4
No	86	43.6
**Total**	194	100
**Has the child been tested for HIV?**		
Yes	188	96.9
No	6	3.1
**Total**	194	100
**The child’s HIV status at 6 weeks**		
Positive	-	-
Negative	194	100
**Total**	194	100

## Discussion

**Implications for maternal and infant healthcare:** according to the results of the pregnancy intention screening, a sizable percentage of respondents who experienced unintended or unexpected pregnancies were more likely to be at risk for experiencing maternity care delays, which can harm a pregnancy's and the baby's postnatal outcomes. Women should be risk-screened and advised about the effects of an unsuccessful pregnancy. Preconception counseling aims to improve both direct and indirect prevention, treatment adherence, and general well-being before becoming pregnant [4,10-14]. Monitoring and controlling viral loads with ART (Anti-Retroviral Treatment) has the benefit of improving maternal health and reducing MTCT [15].

**Impact of viral load monitoring and suppression on maternal and infant health:** when viral load is monitored and suppressed, low birth weights, stillbirths, mortality, and morbidity rates are reduced [16-19]. The reasons why only a small percentage of respondents had viral loads exceeding one thousand could be related to their inability to follow their treatment plans or their complete absence from ARV medication. It makes sense that more effort should be made to ensure that even fewer percentages of HIV-positive women of childbearing potential who want to conceive have undetectable viral load measurements given that a decline in viral loads is linked to a decrease in HIV MTCT and infant and maternal mortality rates.

**Effect of nutritional services and HIV testing on maternal and infant health decision-making:** only a small portion of respondents (approximately a quarter) did not obtain nutritional services, according to the findings of nutritional services. Malnourished women are more likely to have vitamin deficiencies, which raises the risk of preterm labor and low birth weight fetuses [20-24]. Several maternal and neonatal risk factors, including a higher likelihood of diabetes mellitus, high blood pressure, and cardiomyopathy in the fetus, as well as a higher risk of difficult births, C-sections, and delivery problems in the mother, have been linked to obesity [3,7,25-27]. All the infants who had been tested returned negative results. Women's unique decisions regarding pregnancy and deliveries are typically influenced by a complex interplay of circumstances, ranging from the availability and access to information, and the accessibility of health facilities, to personal preferences and social predispositions. These women's HIV testing results make the decision-making process even more difficult. Although little is known about how prenatal care and HIV testing affect women's desire to become pregnant, as antiretroviral medication becomes more accessible, HIV-positive women are living longer and in better health.

**Limitations:** unexpectedly, the research had challenges, such as the COVID-19 Pandemic, even though it started while data was being gathered. Data collection took longer because respondents were harder to reach than they were before the outbreak. Additionally, several respondents could not read or write; therefore, they required assistance from study assistants to complete the surveys.

**Interpretation:** this study focused on pregnancy intention screening, viral load measurement and suppression, and nutritional services in the context of preconception care. It revealed that preconception care services are limited in Nyeri County, with prophylaxis being a notable aspect that yielded positive infant health outcomes.

**Generalizability:** the external validity of this study depends on several factors, including the prevalence of HIV in other counties, the availability and accessibility of preconception care services, and the cultural and social factors that might affect the uptake and effectiveness of these services in different contexts.

## Conclusion

In conclusion, the study significantly contributes to the understanding of preconception care challenges in Nyeri County, offering insights into gaps in family planning, viral load management, and nutritional services. These contributions can guide the development of targeted interventions to improve maternal and child health outcomes in the context of HIV care.

### What is known about this topic

- *Preconception care encompasses a variety of services, including medical assessments, optimization of HIV treatment, management of contraception, and guidance on lifestyle and nutrition;*
- *The implementation of preconception care services has a beneficial influence on preventing mother-to-child transmission (MTCT) of HIV;*
- *A well-executed preconception care strategy plays an important role in minimizing the risk of HIV transmission to infants; by combining antiretroviral therapy with caesarean section delivery, effective preconception care can significantly reduce the transmission risk to less than 1%; this emphasizes the importance of a coordinated and targeted approach to preconception care in achieving optimal outcomes for both mothers and infants*.

### What this study adds

- *The study highlights a significant gap in pregnancy intention screening, with only 33.7% of respondents planning their pregnancies; the statistical link between educational attainment and planned conception emphasizes the pivotal role of education in informed family planning decisions;*
- *The study sheds light on the limited viral load assessments before conception among HIV-positive respondents, emphasizing a potential risk of mother-to-child HIV transmission;*
- *The study evaluates the provision of nutritional services in Nyeri County and identifies areas for improvement, such as the low percentage receiving nutrition counseling and supplements*.
